# Enchiridion Medicum, or Manual of Practical Medicine, the Result of Fifty Years' Experience

**Published:** 1844-10-01

**Authors:** 


### ENCHIRIDION MEDICUM, OR MANUAL OF PRACTICAL MEDICINE; THE RESULT OF FIFTY YEARS' EXPERIENCE. Translated from the German of *M. Hufeland*, by Drs. *Bruchhausen* and *Nelson*. Baillier, London, 1844.

IF every physician were sworn, on taking out his diploma, to register each case and observation, most carefully and scrupulously, and deposit the manuscript in a building for public inspection, the Archives of that Temple would be worth recording a century after its erection! The medical practitioner would there find many a beacon to warn him from falling among rocks, shoals, and quick-sands, as well as to curb his pride in thinking himself so often original in his doctrines and practices. As it is, nineteen-twentieths of our hard-earned experience sink into the grave with the perishable remains of the human machine—and all has to be learnt over again by the succeeding generation. The record of experience in books, is of a more questionable character. The idea that the statements are to be laid bare to the public during the life of the author, must naturally have some influence on him, and prove no very powerful inducements for him to publish the "truth, the whole truth, and nothing but the truth," seeing that he had taken no oath to do so, and that his kind professional brethren would not be over-anxious to cover over his faults, and hide them from the world at large. SELF-LOVE has, no doubt, brought into the world a large brood of fair-looking urchins externally; but who are lob-sided and rickety when stripped of their feathers. TIMIDITY or modesty, on the other hand, has kept in the back-ground many a production that would have done great service to the community at large, and much honour to its parent. The venerable Hufeland has adopted the plan of bequeathing to his country, and to posterity, the results of half a century's experience—in a volume containing a vast deal of valuable matter, intermixed, however, with much that is antiquated and now obsolete. The work is curious, also, as exhibiting a fair specimen of German theory and practice for the last forty or fifty years—and from which many useful hints may be taken by the wisest and oldest among us in these islands. In this volume German minuteness is extremely well characterized. A first-rate galloping physician of this metropolis would be a little startled to have his fifteen or twenty minutes' consultation broken in upon, or rather entirely occupied in questions respecting the plethoric, phlogistic, sthenic, adynamic, weak, feeble, nervous, dry, lax, spongy, lymphatic, gastric, bilious, atrabilious, rheumatic, catarrhal, psoric, venous, hæmorrhoidal, phthisical, apoplectic CONSTITUTION of his patient! Yet all these distinctions are minutely discussed by Hufeland, before he enters on the hereditariness, sex, age, periods, temperaments, idiosyncracies, habits, &c. &c. of the sick man or woman! And, after all these are mastered, the student finds himself merely on the first step of a long flight that leads to the temple of investigation! PATHOGENESIS, or the origin of the disease, occupies large space, and is divided into numerous heads—then comes symptomatology; one item of which, the PULSE alone, would require a long life and wide experience to become acquainted with! The pulse! ye gods, what a variety has come under the TACTUS ERUDITUS of the venerable author! That phenomenon, symbol, or action which varies with every emotion of the patient's mind, and which is now, as in the days of Hippocrates, "*res fallacissima,*" is traced by Hufeland through all its Proteian shapes, till he and we are lost and bewildered in the trackless path! But we must give up the attempt even to enumerate the names of the various pulses, together with countless other branches of symptomatology, though we recommend their perusal, and even study, to the young practitioner, as useful tasks in leisure hours.

We shall only be able to glance at some of the peculiarities of this volume, as characterising German practice, and differing from English. There are many most admirable and judicious moral precepts laid down in this volume. The following remark ought to be written in letters of gold, and hung up in the library of every medical practitioner in this country.

"It is revolting to hear of physicians, who know the difficulties of the art and of forming opinions regarding it, judge their colleagues with severity, harshness, contempt, or disclose their faults, and try to raise themselves by lowering others.

"Oh! that I were able to impress the minds of my brethren with the truism, as forcibly as I am penetrated by it: He who degrades a colleague, degrades himself and his art. For, in the first place, the more the public becomes acquainted with faults of physicians, the more will physicians become exposed as contemptible and suspicious, and the more will such exposure impair confidence." 14.

Hufeland does not regard *consultations* with a very favourable eye—especially when they are numerous. They have long appeared problematical to us. If patients knew their own good, they would be chary in congregating three or four doctors around their bed-side. Never was axiom more false than the hacknied one, viz. "in the multitude of counsel," &c. There is far more folly than wisdom in the procedure! One individual generally dictates the treatment; or, if not, there is wrangling or confusion. There ought never to be more than two consultants, and he who is called in last, should generally have a carte-blanche for trying *his* plan for a few days; when, if no improvement results, the original consultant may fairly urge the treatment he had been previously pursuing, or suggest some plan different from both.

In respect to FEVER—Hufeland avers that there is only "one acute disease—fever." Every fever is of an inflammatory nature, and liable to generate real local inflammation. Every fever, in the same individual, can be converted into all the different species of fever. Every fever is "indispensably accompanied with increased generation of *heat*." Hufeland ought to have known that many fevers extinguish life with the heat below par.

Hufeland does not agree with Broussais or Clutterbuck in ascribing fever to topical inflammation of brain or bowels. He believes that local inflammations are often the consequences or complications of fevers, rather than the causes of them. Although he considers all fevers as one in their nature, he divides them practically into several species. The Pathogenesis, or Etiology, he considers as the chief cause of variety in fevers. Thus, "violent fright creates a nervous fever—violent anger a bilious fever—excesses in wine, &c. an inflammatory fever—putrid substances or contagion a putrid fever." A prevailing epidemic will swallow up all distinctions, and amalgamate all forms of fever.

In Therapeutics, Hufeland has evidently been a temporizing physician—avoiding rough remedies, and acting pretty much on the "*medicine expectante*" principle. Although he considers all fevers as essentially phlogistic, he does not dream of cutting them short by heroic treatment.

"Every acute fever is a phlogistic state of the body, and consequently the remedial means called for is antiphlogistic. Therefore, in the commencement, and as long as the character of the fever is not established, the antiphlogistic treatment is the best.

"Further, we must never forget, that in every acute fever the healing principle and the vital power are one and the same thing; nay more, fever itself is nothing else than a curative process which brings about critical alterations, termination, and a restoration from disturbance to healthy equilibrium,—yea, in many cases, Nature uses no other means than fever to cure disease. Therefore, the office of art is by no means to remove fever itself, but solely to guide its operation in such a manner, that it attains the end of effecting a perfect crisis; art can do no more than to clear away obstacles which oppose it, to moderate the vital power, when too violently excited, to raise and strengthen it when too weak; in short, to confine it within that medium degree of activity, which alone can effectuate a critical operation." 82.

Essentials in fever are mental and corporeal quiet—horizontal position—abundance of aqueous drink—absence from food. Nature herself points to these indications—and it is highly probable that these alone would conduct to as safe a termination as the most complicated system of polypharmacy! This, however, must not be told in Gath!

Our author considers acute rheumatism and bronchitis as the same inflammation, and only differing as to seat. Surely this is not correct pathology. The tissues implicated in the two inflammations are anatomically and physiologically different, and the treatment itself is very different. Hufeland's therapeutics in acute rheumatism are extremely inert, and colchicum or mercury is never alluded to! "The fundamental idea, says he, of cure, is restoration of the cutaneous action." Yet in acute rheumatism, there is generally a great deal too much perspiration. He makes one good remark not sufficiently attended to in this country, viz.—"Too large or unnecessary abstractions of blood will tend to protract rheumatism." They will do more—they will, too, induce metastasis to the heart or other organs. The same may be said of warm baths. They are dangerous both in articular and pulmonary inflammations. He makes no allusion to evaporating lotions in acute rheumatism.

The *Oriental Cholera* he conceives to arise from specific miasmata in the East, and spread by *infection* to the westward. Venesection and an emetic are the primary remedies—calomel and cold water afterwards.

*Rabies Canina* arises from "poisoning of the nerves." Prophylaxis alone can be depended upon. "Scarification, cupping, and cauterizing the wound by gunpowder." He mentions not excision, which is the most effectual of all means, when it can be performed.

*Intermittent Fever* ought not to be stopped suddenly, "since something critical must be attributed to it." Emetics, purgatives, and such like, must be tried, before cinchona can be ventured on. Arsenic is considered too dangerous a drug to be ever ventured on, even in the most obstinate quartans. This is a great error.

*Pneumonia.*—Hufeland appears to have little or no faith in auscultation. He merely alludes to it in this important class of complaints as an illusory means of diagnosis, by which alone the existence of pulmonary diseases can never be discovered! Here is a remarkable proof that long experience does not always secure accuracy of diagnosis or efficacy of practice. "Venesection, tartar emetic, and vesicatories are the principal remedies." "Weak tuberculous lungs, and phthisical disposition strongly call for abstraction of blood." He recommends the patient to be bled from both arms, in the horizontal position, till the pulse falters or becomes intermittent. This is bold, and somewhat dangerous practice. If syncope take place in such a posture, there may be some difficulty in restoring the circulation; whereas, if it occur in the upright posture, the patient immediately recovers by laying him horizontal.

*Carditis.*—This important disease occupies about three-quarters of a page. There is no allusion to metastasis—to the stethoscope—to percussion—to effusion into the pericardium—nor to any of the sounds emitted during inflammation of the heart and its membranes. Neither is there mention of calomel and opium as auxiliaries to other means! The disease is to be treated as pneumonia.

*Epilepsy.*—In this opprobrium medici there is no mention of nitrate or oxide of silver. Oxyd of zinc is the favourite remedy of Hufeland. Chorea is treated in the same way, and purgatives are overlooked.

We need not continue to point out farther the peculiarities of German practice. They differ from the practice and opinions of these isles, in almost every disease. The general character, however, of continental therapeutics is inefficiency, as compared with ours. We are ready to admit that, in many cases, the inertness of German therapeutics, and the medicine expectante of France, are safer measures than the heroic practice of "Young England," as regards bleeding, purging, and mercury. But, in many other cases, precious time is lost which cannot afterwards be regained. Although there is a good deal of what we might term unnecessary minuteness in Hufeland's volume, the work contains a vast deal of accurate and important observations, which even the old practitioner may peruse with advantage, and the young refer to as a hand-book or inexhaustible magazine of valuable materials.

